# Effect of metformin as an adjuvant therapy to letrozole on estradiol and other biomarkers involved in the pathogenesis of breast cancer in overweight and obese postmenopausal women: a pilot study

**DOI:** 10.1007/s00228-022-03444-6

**Published:** 2022-12-23

**Authors:** Aya Ahmed El-attar, Osama Mohamed Ibrahim, Suzan Ahmed Alhassanin, Enas Said Essa, Tarek Mohamed Mostafa

**Affiliations:** 1 Department of Clinical Pharmacy, Faculty of Pharmacy, Tanta University, Tanta, 31527 Egypt; 2 Department of Oncology and Nuclear Medicine, Faculty of Medicine, Menoufia University, Menoufia, Egypt; 3 Department of Clinical Pathology, Faculty of Medicine, Menoufia University, Menoufia, Egypt

**Keywords:** Metformin, Letrozole, Breast cancer, Estradiol, Osteocalcin, Leptin

## Abstract

**Introduction:**

Metformin may provide a therapeutic benefit in different types of malignancy.

**Purpose:**

We aimed at evaluating the effect of metformin as an adjuvant therapy to letrozole on estradiol and other biomarkers involved in the pathogenesis of breast cancer in overweight and obese postmenopausal women.

**Methods:**

Seventy-five postmenopausal stages II–III breast cancer female patients were assessed for eligibility in an open-labeled parallel pilot study. Forty-five patients met the inclusion criteria and were assigned into three arms: the lean arm (*n* = 15) women who received letrozole 2.5 mg/day, the control arm (*n* = 15) overweight/obese women who received letrozole 2.5 mg/day, and the metformin arm (*n* = 15) overweight/obese women who received letrozole 2.5 mg/day plus metformin (2000 ± 500 mg/day). The intervention duration was 6 months. Blood samples were obtained at baseline and 6 months after intervention for the measurement of serum estradiol, leptin, osteocalcin levels, fasting blood glucose concentration, and serum insulin.

**Results:**

After the intervention and as compared to the control arm, the metformin arm showed a significantly lower ratio to the baseline (significant reduction) for estradiol (*p* = 0.0433), leptin (*p* < 0.0001), fasting blood glucose (*p* = 0.0128), insulin (*p* = 0.0360), osteocalcin serum levels (*p* < 0.0001), and the homeostatic model assessment of insulin resistance “HOMA-IR” value (*p* = 0.0145). There was a non-significant variation in the lactate ratio to the baseline among the three study arms (*p* = 0.5298).

**Conclusion:**

Metformin may exert anti-cancer activity by decreasing the circulating estradiol, leptin, and insulin. Metformin might represent a safe and promising adjuvant therapy to letrozole in overweight/obese postmenopausal women with breast cancer.

**Trial registration:**

ClinicalTrials.gov Identifier: NCT05053841/Registered September 23, 2021 - Retrospectively.

## Introduction

Breast cancer is the most common cancer in women worldwide [1]. About two thirds of estrogen and/or progesterone receptors are expressed in about two thirds of breast tumors. Consequently, blocking estrogen signaling in cancer cells is essential during the treatment of breast cancer [2].

In premenopausal females, most of the estrogen is synthesized by the ovaries. On the other hand, following menopause, the major estrogen source is the extra ovarian adipose tissue where the androstenedione and testosterone are converted to estrone and estradiol [3, 4].

Obesity is considered a risk factor for cancer and cancer-related mortality [5]. Obesity is usually associated with poor prognosis, particularly in postmenopausal women with breast cancer. Despite the real mechanisms that are not completely understood, obesity is accompanied by elevated estradiol level, a well-recognized postmenopausal breast cancer risk factor [6]. Moreover, the inflammatory signaling reported within the breast tissue of obese females extensively promotes estrogen signaling, principally by changing the aromatase enzyme expression which in turn increases estrogen production and induces tumor progression [7].

The endocrine therapies available for the management of breast cancer include selective estrogen-receptor modulators (tamoxifen), a selective estrogen-receptor degrader (fulvestrant) as well as aromatase inhibitors (AIs). Aromatase inhibitors hinder aromatase enzymes from converting androgens to estrogens with a subsequent decrease in estrogen concentration. Third-generation aromatase inhibitors include the steroidal irreversible inhibitor (exemestane) and the non-steroidal reversible inhibitors (letrozole and anastrozole) [8]. Nowadays, aromatase inhibitors are given at the same dose regardless of body weight or body surface area [9]. However, there is evidence for the differential effects of anastrozole in overweight or obese women versus normal-weight postmenopausal women with breast cancer [10].

Metformin is the first-line treatment for type 2 diabetes, and it could be used in the management of polycystic ovarian syndrome [11, 12]. Furthermore, some studies revealed that patients with a variety of cancers may get therapeutic benefits from metformin [13]. The anti-tumor activity of metformin could be linked to its negative effects on metabolism [14, 15].

In this context, our research aimed at evaluating the effect of metformin as an adjuvant therapy to aromatase inhibitor (letrozole) on estradiol and other biomarkers involved in the pathogenesis of breast cancer in overweight and obese postmenopausal women.

## Patients and methods

### Study design, patients’ population, and treatment allocation

The study design was a controlled open-labeled parallel pilot study. The final analysis of this study involved 45 overweight/obese postmenopausal women with stage II and stage III breast cancer who were recruited from the Clinical Oncology Department, Menoufia University Hospital, Menoufia, Egypt. Tanta University National Research Ethics Committee approved the current study (approval code: 34653/4/21). The study was carried out following the ethical standards of the Declaration of Helsinki in 1964 and its later amendments. The study was registered at ClinicalTrials.gov with ID: NCT05053841. All participants gave their informed consent.

The inclusion criteria were eligible aromatase inhibitor treatment-naïve lean and overweight/obese postmenopausal women with stage II and stage III breast cancer who were assigned to receive hormonal therapy with letrozole. Postmenopausal is defined as age ≥ 55 years old and 1 year or more of amenorrhea or age < 55 years old and 1 year or more of amenorrhea, with an estradiol level less than 20 pg/ml [16]. The inclusion criteria also included overweight women (BMI ≥ 25 kg/m^2^ and < 30 kg/m^2^), obese women (BMI ≥ 30 kg/m^2^), and non-obese women (BMI between 18 and 25 kg/m^2^). The exclusion criteria were diabetic women, women with metabolic syndrome, women with last menstrual cycle less than 1 year ago, and patients with any disorder that increases the risk of acidosis including heart failure, renal failure, and chronic obstructive pulmonary disease (COPD), and women who were treated with luteinizing hormone-releasing hormone agonists (LHRH).

The randomization was done using the sealed envelope method. This simple randomization method is easy to implement in clinical research. However, in a relatively small sample size clinical research, this method can result in an unequal number of participants between arms. Patients in the lean arm (*n* = 16) were non-obese postmenopausal women with breast cancer who received letrozole 2.5 mg/day for 6 months. Thirty-seven overweight/obese postmenopausal women with breast cancer were randomly assigned to either the control arm (*n* = 19) or metformin arm (*n* = 18) who received letrozole 2.5 mg/day for 6 months as in the lean arm. However, the patients in the metformin arm received the letrozole regimen as the lean and control arms plus metformin 2000 ± 500 mg/day for 6 months. The metformin dosage was slowly titrated upward, beginning with 500 mg/day through the first week and then titrated up by 500 mg every week till achieving the final dose which was determined according to the BMI of each participant [17]. Women with BMI < 30 kg/m^2^, BMI ≥ 30 kg/m^2^, and BMI ≥ 35 kg/m^2^ were given 1500, 2000, and 2500 mg/day of metformin respectively. The study duration was 6 months since the favorable effect of metformin on estradiol level was reported to be achieved within 6 months independently of BMI [18]. All patients were recommended to decrease their carbohydrate intake in the evening, but none of them followed the recommended dietary plan.

All patients included in the study had stage II and stage III breast cancer disease with lymph node involvement. The patients were included in the study after finishing their treatment with chemotherapy/surgery/radiotherapy. The chemotherapy regimen used for all participants was fluorouracil, epirubicin, and cyclophosphamide (FEC regimen) for 3 cycles (cycle every 21 days) followed by paclitaxel for 3 cycles (9 weeks). After finishing the chemotherapy cycles, all patients were submitted to surgery by modified radical mastectomy (MRM). Within 3–4 weeks after surgery, all patients were submitted to radiation therapy (5 sessions every week for a total of 16 sessions) and started on hormonal therapy with letrozole.

It is worth mentioning that there were six patients with positive HER2 in the three study arms (two patients in the lean arm, one patient in the control arm, and three in the metformin arm). Those six patients had low HER2 (positive 1 or 2) with immunohistochemistry (IHC), and the confirmatory analysis through fluorescence in situ hybridization (FISH or ISH) revealed negative HER2 (HER2-negative). Consequently, those six patients did not receive targeted therapy with trastuzumab [19, 20].

### Demographic, clinical, and anthropometric data

Following enrollment, clinical and demographic data were collected including age, stage of the disease, and receptor status. Furthermore, all participants were subjected to anthropometric measurements, including weight, height, and body mass index (BMI = weight (kg)/height^2^ (m)) calculated at baseline and 6 months after the assigned treatment.

### Blood sample collection

At baseline and 6 months after the intervention, blood samples were withdrawn after overnight fasting from the antecubital vein into plain venipuncture tubes. Blood samples were allowed to coagulate and then centrifuged for 10 min at 3000 rpm. The separated sera were stored at − 80 °C until analysis of the biological markers (fasting insulin, estradiol, osteocalcin, leptin, and lactate levels).

### Biochemical analysis

Fasting blood glucose levels were determined by the glucose oxidase method (Spinreact, Spain; Catalogue No.: MD41011). Fasting insulin level was assayed using an enzyme-linked immunosorbent assay (ELISA) kit (Diagnostic Automation/Cortez Diagnostics, Inc., USA: Catalogue No.: 1606–15). The HOMA-IR index was used to estimate insulin resistance (IR), which is defined as fasting insulin level (IU/ml) times fasting blood glucose (mg/dl) divided by 405 [21]. Enzyme-linked immunosorbent assay kits were used to measure the serum estradiol level (DRG International, Inc., USA; Catalogue No.: EIA-4399), serum human osteocalcin level (Epitope Diagnostics Inc., San Diego, USA; Catalogue No.: KT 809) and serum leptin level (Diagnostic Biochem Canada Inc., Ontario, Canada, Catalogue No.: CAN-L-4260). Serum lactate was determined by the colorimetric method (Spinreact, Spain; Catalogue No.: 1001330).

### Assessment of patients’ adherence and drug safety

Every month, patients were closely monitored to assess their compliance and to report any adverse reactions to the study medications. Each woman in the three study arms was asked to return the empty tablet strips at the end of each month to ensure her adherence. Adherence to medications was estimated using the medication refill rate (percentage of drug coverage = number of days in the period covered/number of days in the period that should be covered). Metformin’s safety was evaluated by asking the patients about abdominal pain, loss of appetite, diarrhea, nausea, vomiting, and other adverse effects. The adverse effects were reported using the adverse effects reporting form and from the patients’ sheet. The National Cancer Institute Common Terminology Criteria for Reporting Adverse Events (NCI-CTCAE; Version 5, 2017) was used for grading the reported adverse effects.

### Sample size

Although the sample size used in the current study is small, it exceeds the suggested sample size of 12 per arm, which was reported for a pilot study [22].

### Statistical analysis

Analysis of variance (ANOVA) test and Kruskal–Wallis test followed by a post hoc test were applied to test the significant difference in the measured parameters and their ratio to baseline among the three studied arms. The paired Student’s *t*-test was used to compare the mean values at baseline and the mean values obtained 6 months after treatment within the same arm. The chi-square test was used to compare the categorical data as well as to analyze the reported adverse effects. Values were presented as mean ± standard deviation (SD) and median (interquartile range) for quantitative variables and as numbers and percentages for qualitative variables. The data were coded and inserted using Version 7.0 of GraphPad Prism (GraphPad Software, San Diego, CA, USA). All *p*-values were two-tailed, and a *p*-value < 0.05 was considered significant.

## Results

**Fig. 1:**
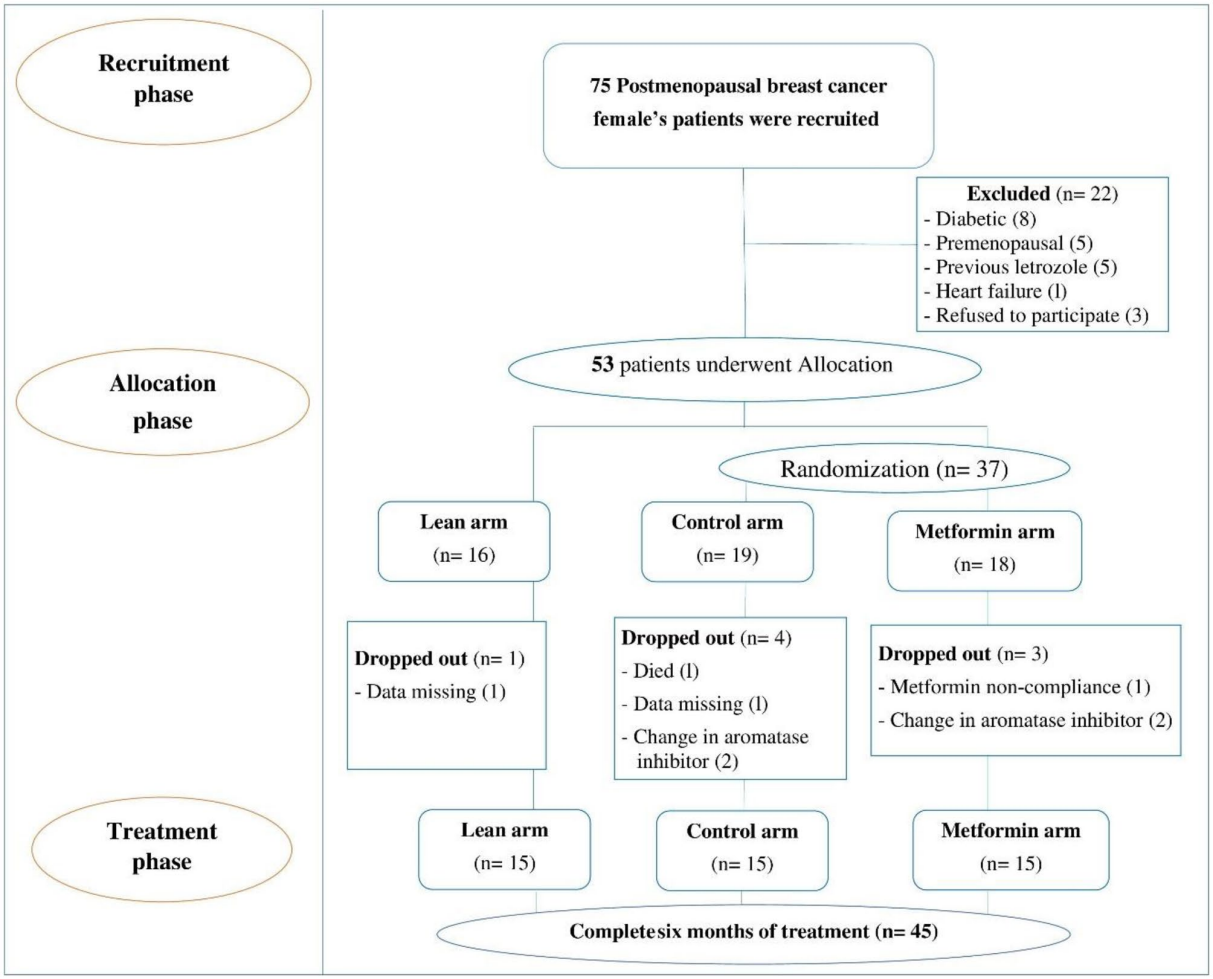
Flow chart of the study participants

During the current study, 75 postmenopausal women with stage II and stage III breast cancer were screened for eligibility; 22 women were excluded, and 53 women were allocated to the three study arms. Out of those 53 patients, 16 were lean (the lean arm), and 37 were overweight or obese patients that were then further randomized into the control arm (*n* = 19) and the metformin arm (*n* = 18). During the study course, 8 women in the three study arms dropped out due to missed data, death, non-adherence, and a change from letrozole to another aromatase inhibitor, and consequently, their preliminary data were omitted from the final analysis. Therefore, only 45 patients (15 patients in each arm) completed the study. The flow chart of the study participants is shown in Fig. 1.

### Demographic, clinical, and anthropometric data

At baseline, all arms were statistically similar regarding age, height, stage of breast cancer disease, and receptor status. All patients received the same chemotherapy (FEC/paclitaxel) and underwent the same surgery, a modified radical mastectomy (MRM). Starting from the confirmatory diagnosis of the disease and initiating treatment till the end of radiotherapy sessions, there were non-significant variations in the duration of breast cancer disease among the three studied arms (*p* = 0.9054).

According to the selection criteria, there was a statistically significant variation among the lean arm and both the control and metformin arms concerning body weight and BMI (*p* < 0.001). However, body weight and BMI showed non-significant variations between the control and metformin arms at baseline. The baseline demographic, clinical, and anthropometric data of the study participants are demonstrated in Table 1.

**Table 1:** Baseline demographic, anthropometric, and clinical data of the study participants

**Variables**	**Lean arm (*n*** **= 15)**	**Control arm (*n*** **= 15)**	**Metformin arm (*n*** **= 15)**	***p*-value**
**Age (years)**	51 ± 6.74	53.33 ± 7.68	53.67 ± 5.49	0.499
**Weight (kg)**	59.73 ± 3.24	87.13 ± 12.99	91.87 ± 17.57	< 0.0001^*^
**Height (cm)**	157.7 ± 2.46	155.9 ± 3.54	157.9 ± 7.04	0.462
**BMI (kg/m**^**2**^**)**	24.01 ± 1.2	35.86 ± 5.35	37.02 ± 7.71	< 0.0001^*^
**Breast cancer stage**				0.554
**Stage II A**	3 (20%)	1 (6.667%)	4 (26.667%)	
**Stage II B**	2 (13.333%)	3 (20%)	3 (20%)	
**Stage III A**	5 (33.333%)	6 (40%)	5 (33.333%)	
**Stage III B**	2 (13.333%)	1 (6.667%)	3 (20%)	
**Stage III C**	3 (20%)	4 (26.667%)	0 (0%)	
**Receptor status**				0.8979
**ER-positive**	15 (100%)	14 (93.33%)	14 (93.33%)	
**PR-positive**	14 (93.33%)	15 (100%)	14 (93.33%)	
**HER2 (low HER2) positive 1 or 2**	2 (13.33)	1 (6.67%)	3 (20%)	
**FISH test for HER2**	Negative	Negative	Negative	
**Duration of breast cancer disease (weeks)**	34.73 ± 2.915	35.07 ± 2.89	34.6 ± 3.043	0.9054

Data are presented as mean ± SD, number, and percentage. Chi-square test was used for categorical data and ANOVA test was applied for continuous data

*BMI* body mass index, *ER* estrogen receptor, *PR* progesterone receptor, *HER2* human epidermal growth factor receptor 2, *FISH* fluorescence in-situ hybridization

*Significant difference (*p* < 0.05)

**Fig. 2:**
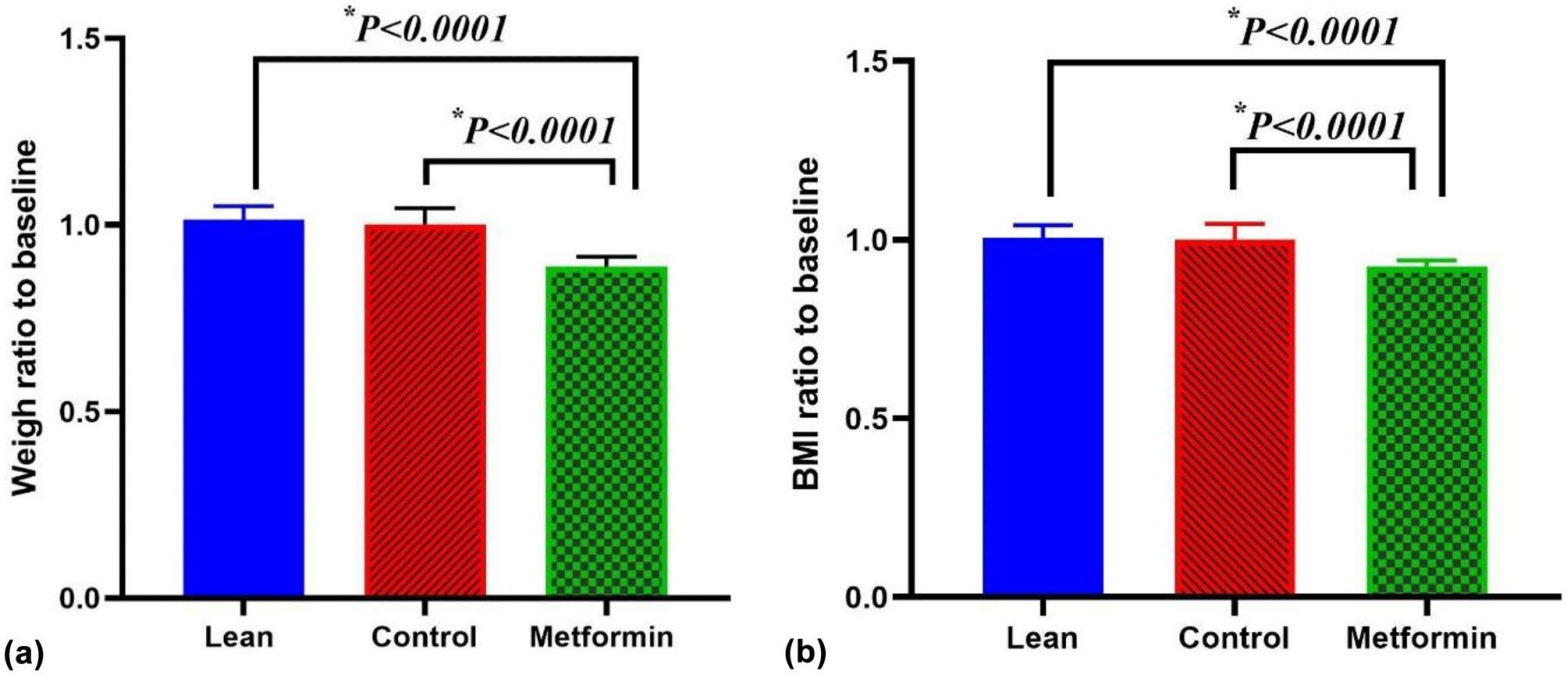
Ratio to baseline data for weight (**a**) and BMI (**b**) among the three arms throughout the treatment course

After the intervention and compared to the control and lean arms, the metformin arm showed a significantly lower ratio to the baseline (significant reduction) of body weight (*p* < 0.0001 and *p* < 0.0001, respectively) and BMI (*p* < 0.0001 and *p* < 0.0001, respectively). The ratio to the baseline of body weight and BMI for the three studied arms is depicted in Fig. 2.

### Effect of intervention on glycemic parameters

Fasting blood glucose levels revealed non-significant variations between the control and metformin arms at baseline. However, the previously mentioned arms and the lean arm showed a statistically significant variation (*p* = 0.0266 and *p* = 0.0049, respectively). Six months after therapy, both the metformin and lean arms showed significantly lower mean fasting blood glucose levels than the control arm (*p* < 0.0001 and *p* = 0.0373, respectively). Furthermore, the metformin arm showed significantly lower fasting blood glucose levels compared to the lean arm (*p* = 0.0232).

Concerning the fasting blood glucose ratio to baseline, the metformin arm showed a significantly lower fasting blood glucose ratio to baseline compared to both the control arm and the lean arm (*p* = 0.0128 and *p* = 0.0015, respectively). However, there was no statistically significant difference in the ratio to baseline between the control arm and the lean arm (*p* > 0.9999).

At baseline, both the control and metformin arms showed non-significant variations in fasting insulin levels. However, both arms showed significant elevation in fasting insulin levels compared to the lean arm (*p* = 0.0415 and *p* = 0.0401, respectively). Six months after the intervention, both the metformin and the lean arms revealed a significant decrease in the mean level of fasting insulin as compared to the control arm (*p* = 0.0042 and *p* = 0.0037, respectively). Moreover, there was a non-significant variation in the mean value of fasting insulin between the metformin and lean arms (*p* = 0.9991).

Metformin significantly reduced the insulin ratio to baseline compared to the control arm (*p* = 0.0360). Moreover, there was a non-significant variation in the mean ratio to the baseline of fasting insulin between the lean arm and both the control and metformin arms (*p* = 0.8305 and *p* = 0.1274, respectively).

Both the control and metformin arms had non-significant variations in HOMA-IR values before treatment initiation. However, the control and metformin arms showed significantly higher HOMA-IR values than the lean arm (*p* = 0.0118 and *p* = 0.0067, respectively). Six months after the intervention, both the metformin and lean arms showed significantly lower mean HOMA-IR values than the control arm (*p* = 0.0008 and *p* = 0.0031, respectively). Additionally, a non-significant decrease in the mean value of HOMA-IR was observed between the metformin and the lean arm (*p* = 0.8908).

Metformin significantly reduced the HOMA-IR ratio to baseline compared with the control and lean arms (*p* = 0.0145 and *p* = 0.0174, respectively). Additionally, there was a non-significant variation in the mean ratio to the baseline of the HOMA-IR value between the control arm and the lean arm (*p* = 0.9973). Glycemic parameters for the three study arms at baseline and 6 months after intervention are presented in Table 2, and the ratio to the baseline for glycemic parameters is shown in Table 3.

**Table 2:** Biological markers at baseline and 6 months after intervention for the three studied arms

**Variables**	**Lean arm (*n*** **= 15)**	**Control arm (*n*** **= 15)**	**Metformin arm (*n*** **= 15)**	*p-value
**FBG (mg/dl)**				
**At baseline**	83.67 ± 8.347	91.2 ± 5.697	93 ± 8.569	0.0041^*^
**After 6 months**	85.8 ± 5.506	91.87 ± 7.06	79.27 ± 6.829	< 0.0001^*^
**p-**value**	0.3891	0.7690	0.0015^**^	
**Insulin (µIU/ml)**				
**At baseline**	10.24 ± 2.791	14.13 ± 4.722	14.15 ± 4.901	0.0213^*^
**After 6 months**	8.95 ± 1.846	12.53 ± 3.09	8.991 ± 3.375	0.0013^*^
**p-value	0.0925	0.258	0.0003^**^	
**HOMA-IR**				
**At baseline**	2.12 ± 0.6281	3.153 ± 0.9841	3.227 ± 1.13	0.0034^*^
**After 6 months**	1.893 ± 0.425	2.779 ± 0.8742	1.777 ± 0.703	0.0004^*^
**p-value	0.2148	0.236	< 0.0001^**^	
**Estradiol (pg/ml)**				
**At baseline**	4.726 ± 2.07	7.806 ± 3.688	8.056 ± 2.765	0.0049^*^
**After 6 months**	1.519 ± 0.6443	3.571 ± 1.495	2.14 ± 0.713	< 0.0001^*^
**p-value	0.0001 ^**^	0.0003 ^**^	< 0.0001 ^**^	
**Osteocalcin (ng/ml)**				
**At baseline**	12.05 ± 2.168	13.1 ± 2.558	12.03 ± 1.932	0.3362
**After 6 months**	14.49 ± 2.48	16.45 ± 3.168	8.333 ± 2.938	< 0.0001^*^
**p-value	0.0002^**^	< 0.0001^**^	0.0003^**^	
**Leptin (ng/ml)**				
**At baseline**	16.2 ± 9.808	44.58 ± 15.71	56.09 ± 18.59	< 0.0001^*^
**After 6 months**	17.72 ± 8.941	51.6 ± 14.53	24.26 ± 14.14	< 0.0001^*^
**p-value	0.6214	0.1742	< 0.0001^**^	
**Lactate (mg/dl)**				
**At baseline**	19.95 ± 6.116	25.78 ± 8.996	23.65 ± 5.447	0.0829
**After 6 months**	21.41 ± 5.032	24.48 ± 6.844	22.91 ± 7.79	0.4560
**p-value	0.3712	0.4751	0.7629	

Data presented as mean ± SD

*FBG* fasting blood glucose, *HOMA-IR* homeostatic model assessment of insulin resistance

*p-value (comparison of post-treatment data among the three studied arms using ANOVA test)

**p-value (comparison of post-treatment data versus baseline data within the same arm using paired *t*-test)

**Table 3:** Ratio to baseline data of the measured parameters for the three studied arms

**Variables**	**Lean arm (*n*** **= 15)**	**Control arm (*n*** **= 15)**	**Metformin arm (*n*** **= 15)**	***p*-value**
FBG ratio to baseline^d^	1.023^b^ (0.9362- 1.133)	1.000 (0.9406–1.024)	0.8387^a^ (0.7451–0.9753)	0.0010^*^
Insulin ratio to baseline^c^	0.9229 ± 0.2890	0.9987 ± 0.4922	0.6635^a^ ± 0.2378	0.035^*^
HOMA-IR ratio to baseline^c^	0.9610 ± 0.3499^b^	0.9705 ± 0.4925	0.5731 ± 0.2168^a^	0.0069^*^
Estradiol ratio to baseline^d^	0.2539 (0.1758–0.4419)	0.4540 (0.3055–0.6266)	0.2739^a^ (0.2167–0.3197)	0.0286 ^*^
Osteocalcin ratio to baseline^c^	1.216 ± 0.1865^b^	1.269 ± 0.1651	0.6986 ± 0.2666^a^	< 0.0001^*^
Leptin ratio to baseline^d^	1.080^b^ (0.7800–1.209)	1.247 (0.9583–1.593)	0.3966^a^ (0.3314–0.6594)	< 0.0001^*^
Lactate ratio to baseline^c^	1.129 ± 0.3317	0.9932 ± 0.2284	1.023 ± 0.4424	0.5298

Data are presented as either mean ± SD or median (interquartile range)

*FBG* fasting blood glucose, *HOMA-IR* homeostatic model assessment of insulin resistance

*Statistically significant difference among the three arms at *p* < 0.05

^a^Statistically significant in comparison to the control arm at *p* < 0.05

^b^Statistically significant in comparison to the metformin arm at *p* < 0.05

^c^One-way analysis of variance (ANOVA) test was used for comparison between arm with post hoc (Tukey’s multiple comparisons) test

^d^Kruskal–Wallis test was used for abnormally distributed variables with post hoc (Dunn’s multiple comparisons) test

### Impact of intervention on estradiol level

At baseline, there was a non-significant variation in estradiol serum levels between the control and metformin arms. However, both the control and metformin arms showed significantly higher estradiol levels as compared to the lean arm (*p* = 0.0163 and *p* = 0.0088, respectively). After the intervention, the mean estradiol level was significantly lower in both metformin and lean arms as compared to the control arm (*p* = 0.0012 and *p* < 0.0001, respectively). On the other hand, the difference in estradiol levels between the metformin and lean arms was not statistically significant (*p* = 0.2330).

Regarding the estradiol ratio to baseline, the mean estradiol ratio to baseline was significantly lower in the metformin arm as compared to the control arm (*p* = 0.0433). On the other hand, there was a non-significant variation in estradiol ratio to baseline between the lean arm and both the metformin and control arms (*p* > 0.9999 and *p* = 0.0969, respectively). The changes in estradiol serum level at baseline and 6 months after intervention in the three study arms are shown in Table 2, and the ratio to the baseline for estradiol serum level is presented in Table 3.

### Effect of intervention on serum osteocalcin level

Before the initiation of any treatment, osteocalcin levels showed non-significant variation among the three study arms. Six months after treatment, the metformin arm showed significantly lower mean osteocalcin serum concentration than both the control and the lean arms (*p* < 0.0001 and *p* < 0.0001, respectively).

Concerning the osteocalcin ratio to baseline, the metformin arm showed a significantly lower osteocalcin ratio to baseline than the control and lean arms (*p* < 0.0001 and *p* < 0.0001, respectively). However, there were non-significant variations in the mean osteocalcin ratio to baseline between the control arm and the lean arm (*p* = 0.7734). The changes in osteocalcin serum level at baseline and 6 months after intervention in the three study arms are postulated in Table 2 and the ratio to the baseline for osteocalcin serum level is shown in Table 3.

### Effect of intervention on serum leptin level

At baseline, both the control and metformin arms showed statistically similar leptin levels. However, both arms showed significantly higher leptin levels compared to the lean arm (p < 0.0001 and *p* < 0.0001, respectively). After the intervention, both the metformin and lean arms showed significantly lower serum leptin levels than the control arm (*p* < 0.0001 and *p* < 0.0001, respectively). There were non-significant variations in the serum leptin levels between the metformin arm and the lean arm (*p* = 0.3499).

Regarding the leptin ratio to baseline, the metformin arm showed a significantly lower leptin ratio to baseline than both the control arm and the lean arm (*p* < 0.0001 and *p* = 0.0007, respectively). There were non-significant variations in the mean leptin ratio to baseline between the control arm and the lean arm (*p* > 0.9999). The changes in leptin serum level at baseline and 6 months after intervention in the three study arms are presented in Table 2 and the ratio to the baseline for leptin serum level is shown in Table 3.

### Effect of intervention on serum lactate level

At baseline and 6 months after the intervention, mean serum lactate levels showed non-significant variations among the three study arms. The changes in lactate serum level at baseline and 6 months after intervention in the three study arms are presented in Table 2, and the ratio to the baseline for lactate serum level is shown in Table 3.

### Drug safety and tolerability

All the reported adverse effects were of grades 1 and 2. There were no serious adverse events reported in the three study arms. The most commonly reported adverse effects were hot flushes and gastrointestinal tract related side effects. The incidence of the reported adverse effects was non-significantly higher in the metformin arm as compared to the other two arms (*p* > 0.05). The reported adverse effects are illustrated in Table 4.

**Table 4:** Reported adverse effects of the study medications

**Adverse effect**	**Lean arm (*n*** **= 15)**	**Control arm (*n*** **= 15)**	**Metformin arm (*n*** **= 15)**	***p*-value**
Hot flushes	3 (20%)	4 (26.67%)	5 (33.33%)	0.7111
Vomiting	2 (13.33)	2 (13.33%)	4 (26.67%)	0.5444
Diarrhea	1 (6.67%)	1 (6.67%)	2 (13.33%)	0.7600
Loss of appetite	2 (13.33%)	1 (6.67%)	3 (20%)	0.5616
Heartburn	4 (26.67%)	3 (20%)	5 (33.33%)	0.7111

Data are presented as numbers and percent. Data were analyzed by chi-square test

## Discussion

Estrogen has a key function in the pathogenesis of breast cancer [23]. Aromatase inhibitors (AIs) prevent the conversion of androgens to estrogens by blocking the aromatase enzyme with a subsequent decrease in estradiol [24]. The activity of aromatase enzyme is predominant in adipose tissue and this in turn could lead to the assumption that aromatase activity could be elevated in overweight/obese females. In this context, the clinical activity of aromatase inhibitors in those females may be declined [25]. Metformin is a “star” drug commonly used for type 2 diabetes mellitus. Furthermore, growing evidence demonstrated that metformin may represent a promising chemotherapeutic agent [26]. It may also have a weight-reduction effect on the non-diabetic population through its ability to enhance insulin sensitivity; however, the underlying mechanisms need to be identified [15]. Our research goal was to investigate the effect of adding metformin as an adjuvant therapy to letrozole on estradiol and other biomarkers involved in the pathogenesis of breast cancer in overweight/obese postmenopausal females with stage II and stage III breast cancer.

During the current study, metformin triggered a significant reduction in the mean ratio to the baseline of body weight and BMI; a result seems in consonance with the finding reported by Seifarth et al. [17].

Basic research revealed that long-term exposure to high insulin levels promotes breast cancer progression either directly through insulin receptor isoform A and insulin-like growth factor 1 (IGF-1) receptor activation or indirectly through alternation in the circulating estrogen levels [27]. In the Women’s Health Initiative Observational Study, hyperinsulinemia represented an independently associated risk factor for postmenopausal breast tumors [28]. The results obtained with the current study revealed that 6 months after the intervention, the metformin arm produced a significant decrease in serum insulin level, blood glucose concentration, and HOMA-IR value as compared to the control arm. Our former results seem similar to the findings reported by Meyerhardt et al. [29]. Metformin reduces hyperinsulinemia and hyperglycemia by enhancing hepatic and peripheral insulin sensitivity. It inhibits gluconeogenesis and glucose synthesis in the liver with increasing glucose utilization in the muscles and adipose tissues [30]. The effect of metformin on glucose levels could be mediated through the activation of AMP-activated protein kinase (AMPK) as a consequence of the inhibition of mitochondrial respiratory chain complex I and reduction in the conversion of glycerol and lactate into glucose [31]. Several studies revealed that the intestine may be involved in the metformin blood-glucose-lowering effect. This favorable effect may be attributed to the changes in glucose uptake and anaerobic metabolism of enterocytes and the increase in the synthesis of glucagon-like peptide-1 (GLP-1) [32].

Regarding the results obtained with estradiol, metformin produced a significant decrease in the estradiol levels when compared to the control arm. This effect could be explained by the ability of metformin to reduce the circulating insulin level. Insulin was reported to inhibit the hepatic biosynthesis of sex-hormone-binding globulin (SHBG) with a subsequent increasing the bioavailability of estradiol [33]. The favorable effect of metformin on estradiol may be also attributed to the direct suppressive effect of metformin on aromatase activity [34, 35]. Our result appears to be consistent with the result reported by Campagnoli et al. [36].

The result obtained with osteocalcin 6 months after treatment revealed that metformin significantly reduced osteocalcin levels as compared to the control and the lean arms. There are conflicting reports about the effect of metformin on osteocalcin. Our finding seems compatible with the data published by Roomi et al. [37]. However, Hegazy in 2015 reported that metformin produced a non-significant decrease in the osteocalcin level 12 weeks after treatment and the author concluded that metformin is neither osteogenic nor anti-osteoporotic [38]. On the other hand, Molinuevo et al. reported that metformin administration produced an increase in osteocalcin expression [39]. These conflicting data could be related to the variations in the study protocols, duration of treatment, and the implicated doses of metformin.

A high level of leptin has been linked to both breast tumor aggressiveness and poor prognosis [40]. Leptin was also reported to enhance aromatase expression in MCF7 cell lines and consequently promote the synthesis of estrogen and increase the risk for breast cancer [41]. Our study showed that metformin produced a significant decrease in serum leptin levels as compared to the control arm. However, there was a non-significant variation in mean leptin level between the metformin and lean arms. These results are compatible with the results reported by Annie et al. and Kargulewicz et al. [42, 43]. Although leptin concentration is strictly related to body fat mass, the reduction in leptin levels cannot be completely explained by the weight-reducing effect of metformin since metformin was reported to reduce leptin concentration even in normal-weight subjects [44]. The results obtained with a previously reported in vitro study demonstrated that metformin suppresses mitogen-activated protein kinase (MAPK) activity in adipocytes and consequently can reduce leptin levels [45]. The impact of metformin on leptin concentration may be attributed to the modulation of the hypothalamic leptin receptor gene (ObRb) [46].

The levels of lactate did not change significantly among the three study arms during the study. This result indicates that metformin did not cause lactic acidosis in those patients’ populations. Lactic acidosis is a rare event that takes place with the accumulation of metformin. The risk of lactic acidosis is usually augmented in the elder population and in patients with conditions that predispose them to acidosis such as heart, kidney, and hepatic diseases [47]. Before running the current study, we excluded all women with conditions that predispose them to the development of lactic acidosis including women with heart failure, renal failure, and COPD. The gastrointestinal side effects reported in the current study included diarrhea, nausea/vomiting, and heartburn. The incidence of these gastrointestinal side effects was non-significantly higher in the metformin arm as compared to the other two arms. These aforementioned side effects developed during the early period of the treatment and they were mild and temporary and disappeared with continuous use of medications. In addition, the gastrointestinal side effects were counteracted by taking the study medications after the meal.

The points of strength of the current study include the assessment of many biological markers involved in the pathogenesis of breast cancer, the use of the same brand of metformin and letrozole throughout the study, the use of metformin doses in relation to the BMI of each participant, and the relatively good study duration. However, the current study has some limitations including its designs as a pilot study with a relatively small sample size, the lack of a non-obese arm that receives metformin, and the lack of stratification of patients based on the stage of the disease. In this context, future large-scale and more longitudinal studies involving non-obese arms with stratification of patients based on the stage of the disease are still required.

## Conclusion

Metformin has anti-cancer activity by decreasing the high circulating estrogen level that is linked to the pathogenesis of postmenopausal breast cancer. Metformin is an insulin sensitizer that reduces insulin and glucose levels. Metformin mitigates the high insulin level associated with cancer cell proliferation and poor clinical outcomes. Furthermore, metformin can negatively affect leptin which promotes estrogen biosynthesis. The results obtained from this study may prove the possible safety and tolerability of metformin and advocate that it could represent a promising adjuvant therapy to letrozole for overweight/obese postmenopausal women with breast cancer secondary to its favorable effect on estradiol and other biomarkers involved in pathogenesis of breast cancer. However, large-scale and more longitudinal studies are still needed.

## Data Availability

All data generated or analyzed during the current study are available from the corresponding author upon reasonable request.
